# HTS driven by fluorescence lifetime detection of FRET identifies activators and inhibitors of cardiac myosin

**DOI:** 10.1016/j.slasd.2023.06.001

**Published:** 2023-06-10

**Authors:** JM Muretta, D Rajasekaran, Y Blat, S Little, M Myers, C Nair, B Burdekin, SL Yuen, N Jimenez, P Guhathakurta, A Wilson, AR Thompson, N Surti, D Connors, P Chase, D Harden, CM Barbieri, L Adam, DD Thomas

**Affiliations:** a Photonic Pharma LLC and University of Minnesota, Minneapolis, MN, United States of America; b Bristol Myers Squibb, Princeton, NJ, United States of America

**Keywords:** High-throughput screening, myosin, time-resolved fret, heart failure, allosteric

## Abstract

Small molecules that bind to allosteric sites on target proteins to alter protein function are highly sought in drug discovery. High-throughput screening (HTS) assays are needed to facilitate the direct discovery of allosterically active compounds. We have developed technology for high-throughput time-resolved fluorescence lifetime detection of fluorescence resonance energy transfer (FRET), which enables the detection of allosteric modulators by monitoring changes in protein structure. We tested this approach at the industrial scale by adapting an allosteric FRET sensor of cardiac myosin to high-throughput screening (HTS), based on technology provided by Photonic Pharma and the University of Minnesota, and then used the sensor to screen 1.6 million compounds in the HTS facility at Bristol Myers Squibb. The results identified allosteric activators and inhibitors of cardiac myosin that do not compete with ATP binding, demonstrating high potential for FLT-based drug discovery.

## 1. Introduction

Small molecules that selectively bind to allosteric sites on proteins are highly desirable as drug candidates, but are difficult to identify. Structure-targeted drug discovery —discovery that tracks changes in protein structure —is a promising approach for identifying compounds that bind to specific conformational states of target proteins, altering the protein dynamics and functional landscape. A wide range of spectroscopic approaches have been used to discover small molecule allosteric modulators including single-wavelength fluorescence emission of intrinsic (e.g., tryptophan) and site-specifically attached extrinsic probes, fluorescence excitation and emission spectra, fluorescence polarization, second-harmonic generation, and magnetic resonance [1–4]. Each of these discovery platforms has advantages and disadvantages unique to individual applications. Fluorescence energy transfer approaches that report changes in intermolecular distance between probes has proven particularly useful for interrogating the impact of small molecules on protein allosteric states [2]. The approaches used to detect FRET at high-throughput include emission spectra and ra-tiometric measurements of donor and acceptor emission, which rely on detection of steady-state fluorescence. In contrast, time-resolved fluorescence measurements report the donor excited-state lifetime, on the nanosecond time-scale. Millisecond time-scale time-resolved luminescence measurements using chelated lanthanide probes [5–7] have also been used to monitor protein structure by energy transfer. Both nanosecond-scale fluorescence and millisecond-scale luminescence have long been understood to be exceptionally precise and accurate. Fluorescence probes offer advantages in flexibility and availability, as organic dyes and fluorescent proteins, with nanosecond decay lifetimes, are easily attached to target proteins and offer sensitivity on the nanomolar to micromolar concentration range. Measuring time-resolved fluorescence lifetime (FLT) of nanosecond probes can be a challenge in drug discovery settings. Long-established standard approaches such as time-correlated single photon counting or frequency domain spectrometers are too low-throughput for industrial scale compound library screens.

In a series of previous studies, we established and described the methodology and instrumentation for performing a high-throughput nanosecond time-resolved fluorescence measurement using direct-waveform recording (DWR) [8–12]. The underlying technology transiently digitizes the signal from 10 s of thousands of photons emitted from a single sample following excitation by a single pulse of a microchip laser. This measurement is repeated every 0.1 to 1 ms, thus allowing the time-resolved fluorescence decay of each well in a 1536-well plate to be read as fast as plate-readers can scan the wells. DWR measures nanosecond-scale time-resolved fluorescence de-cays with picosecond-scale precision [8]. Thus small changes in energy transfer corresponding to angstrom-scale in the distance separating donor and acceptor FRET probes can be monitors. Though DWR FLT-detection has been implemented for more than 40 years, initial efforts were limited largely by linearity and precision. It was not until the advent of low-cost, high-bandwidth transient digitizers and highly reproducible low-cost micro-chip lasers, both developed more recently, that DWR FLT-detection in high-throughput applications became practical [8]. We have described key components of this approach and have used it to undertake studies of protein structural kinetics, measuring protein structural changes by time-resolved fluorescence lifetime-detected FRET (FLT-FRET) during a biochemical transient initiated by rapid-mixing [13]. We have also used it to execute small-scale small molecule discovery screens [14–16]. Here we report a collaboration between Photonic Pharma LLC, a company founded to commercialize this approach, and Bristol Myers Squibb to test the effectiveness of this assay platform on an industrial scale, probing a high-profile drug target, cardiac myosin for the identification of allosteric activators and allosteric inhibitors of cardiac contractility. This work is enabled by a well characterized existing allosteric activator (Omecamtiv Mecarbil [OM]) and allosteric inhibitor (mavacamten [Mava]) tool compounds [12, 17] to guide assay development.

Cardiac myosin was chosen for this work because we had previously used DWR-based FLT-detection to characterize allosteric structural transitions associated with cardiac myosin activation and inactivation, using this technology to characterize the structural impacts of small molecule allosteric modulators on these transitions, thus providing proof of concept for executing DWR FLT-HTS to discover novel chemical probes [9, 12]. Cardiac myosin is therapeutically important because it drives contraction in the heart, converting the chemical potential energy of ATP hydrolysis into mechanical work [18]. Cardiac myosin’s working cycle involves coordinating angstrom-to-nanometer scale domain motions with the protein’s ATPase cycle, catalyzed by its active site in coordination with actin-binding, which activates ATPase cycling [19]. We have used spectroscopic probes to track many of these motions and to correlate their movement with individual steps in the myosin ATPase cycle. These studies include direct detection of the allosteric activation of myosin’s lever-arm rotation —the powerstroke that couples ATPase cycling with mechanical work [9, 12, 20]. The myosin powerstroke and its coordination with the ATPase cycle is modulated by multiple allosteric modulators, including blebbistatin [21], OM [17], and Mava [22]. Blebbistatin (Blebb) inhibits actin activation and binds an allosteric site that is adjacent to the ATPase site near the protein’s actin-binding interface [23]. OM activates force generation and enhances cardiac output, binding to an allosteric site located a nanometers away from the ATP binding site, in a region termed the transducer/converter interface [24, 25]. Mava inhibits excessive contraction in the heart muscle by stabilizing the auto-inhibitory state resulting from intramolecular interactions within myosin [23].

The large structural changes associated with movement of the myosin lever-arm, together with the availability of existing clinical compounds that act as allosteric activators (OM) and inhibitors (Mava, Blebb), made cardiac myosin an appropriate choice for our test of a DWR FLT high-throughput screen (HTS) of drug-like small-molecules. The cardiac myosin allosteric FRET sensor was developed in previously published mechanistic studies in laboratories at UMN [9, 12]. We used the existing myosin modulators OM and Mava to validate assay performance through a 60,000-compound pilot screen performed in the BMS screening facility, using instrumentation provided by Photonic Pharma, and then undertook a fully automated 1.6 million-compound screen at BMS to identify new compounds that modulate myosin ATPase activity by changing myosin structure. From this screen, we identified both allosteric activators and inhibitors of cardiac myosin ATPase cycling and calcium activated myofibril ATPase activity.

## 2. Materials and methods

### 2.1. Protein purification and labeling

Bovine cardiac myosin, rabbit skeletal actin, and recombinant bovine ventricular cardiac myosin RLC were purified as described in our previous work [9, 12]. Recombinant RLC was labeled with cysteine-reactive maleimide Alexa-488 dye (Thermo) as described previously [2]. Labeled RLC was exchanged onto purified myosin under high-salt conditions by combining 3 to 4 molar excess of labeled RLC to purified myosin in 50 mM Tris, 600 mM KCl, 2 mM DTT, 12 mM EDTA (pH 7.5) and then incubated for 30 min at 30 °C. We then adjusted the reaction to 12 mM MgCl_2_ and incubated the mixture on ice for 15 min followed by dialysis into 10 mM Tris, 2 mM MgCl_2_ (pH 7.0). Myosin filaments were sedimented by centrifugation and resuspended in 10 mM Tris, 600 mM KCl, 2 mM DTT (pH 7.5) followed by dialysis into 10 mM Tris, 2 mM MgCl_2_ (pH 7.0) and sedimentation by centrifugation. The pellet was resuspended in 10 mM Tris, 600 mM KCl, 2 mM DTT (pH 7.5) and brought to 150 mM sucrose and then snap frozen in 30-microliter droplets in liquid nitrogen prior to long-term storage at − 80 °C. Cardiac myofibrils were purified following protocols established by Solaro [26] and used within three days of preparation.

Smooth muscle actomyosin was prepared from bovine aorta as described previously [27] with modifications. Frozen bovine aortas (Pel Freeze) were thawed overnight in CaPSS buffer (137 mM NaCl, 6 mM NaHCO_3,_ 11.5 mM glucose, 1.7 mM CaCl_2,_ 1.25 mM MgCl2, 5 mM MOPS, 0.1 mM EGTA and 6 mM KCl, pH 7.2) and then rinsed with calcium free PSS (137 mM NaCl, 6 mM NaHCO_3,_ 11.5 mM glucose, 1.25 mM MgCl_2,_ 5 mM MOPS, 0.1 mM EGTA, 6 mM KCl, pH 7.2). Aortas were processed in a meat grinder and homogenized further in a blender. Crude actomyosin was extracted from the homogenate overnight at 4 °C, following the addition of 2.5 ml of extraction buffer (80 mM KCl, 4 mM MgCl_2,_ 4 mM EGTA, 20 mM MOPS pH 7.2, 0.5 mM DTT, 4 mM ATP, pH 7.0) added per gram of homogenized aorta. Following the overnight extraction, the material was centrifuged at 16,000 rcf for 1 h, and the pellet was dialyzed for 18 h using dialysis buffer 1 (125 mM KCl, 5 mM MOPS pH 7.0, 0.5 mM DTT, 0.2 mM EGTA at pH 7.0). The precipitated crude actomyosin was collected by centrifugation at 8000 rcf for 10 min. The centrifugation pellet was dissolved in high ionic strength actomyosin-dissolving buffer (600 mM KCl, 5 mM MOPS pH-7.0, 0.5 mM DTT, 1 mM ATP) and was centrifuged again at 16,000 rcf for 1 hour. The supernatant was dialyzed in 250 mM KCl, 5 mM MOPS pH 7.0, 0.5 mM DTT, 0.2 mM EGTA, 5 mM NaN_3._ Purified actomyosin was collected from the dialysate by centrifugation at 8000 rcf for 10 min. The actomyosin-containing pellet was resuspended in storage buffer (50 mM KCl, 5 mM pH 7 MOPS, 1 mM MgCl_2,_ 1 mM NaN_3_ pH 7.0, 0.5 mM DTT) and flash frozen in liquid nitrogen with 150 mM sucrose for long-term storage at −80 °C.

### 2.2. ATPase assays

#### 2.2.1. Myosin

We measured the MgATPase activity of the purified cardiac myosin using an NADH-coupled assay [9] performed at 25 °C in 2 mM MgCl_2,_ 10 mM Tris (pH 7.5). The reaction mix contained varied concentrations of test compounds with 0.5 micromolar myosin polymerized into filaments by dilution into assay buffer prior to the assay, and 0.2 mM NADH, 0.5 mM phosphoenolpyruvate, 2.1 mM ATP, 10 U/mL lactate dehydrogenase, 40 U/mL phosphorylase kinase. Reactions also contained 1% DMSO or 1% DMSO with varied concentrations of test compounds. We measured time-dependent changes in sample absorbance at 340 nm using a Spectromax Plus microplate spectrophotometer plate reader at 25 °C.

#### 2.2.2. Myofibril

Myofibril ATPase activity was measured using an NADH-coupled assay performed at 25 °C in 35 mM NaCl, 5 mM MgCl_2,_ 1 mM EGTA, 20 mM MOPS (pH 7.0), with free CaCl_2_ at the indicated pCa concentrations. Free Ca 2 + concentrations were calculated using the method of Fabiato and Fabiato [28]. The reaction mix contained 0.05 mg/ml bovine ventricular myofibrils, 0.84 mM phosphoenolpyruvate, 1 U/ml lactate dehydrogenase, 0.5 U/ml phosphorylase kinase, 0.5 mM sodium azide, 0.17 mM NADH, 2.1 mM ATP, dispensed into 384-well assay plates. The absorbance of the reactions was measured at 340 nm for 30 min using a Spectromax Plus microplate spectrophotometer plate reader at 25 °C.

#### 2.2.3. Aorta smooth muscle actomyosin ATPase

Smooth muscle actomyosin ATPase was measured using a malachite green-based phosphate detection endpoint assay [29] performed in a 96 well plate using the malachite Green (MG) Phosphate Assay Kit (Sigma-Aldrich). MG working solution was prepared by mixing 100 vol of assay kit reagent A with 1 vol of assay kit reagent B. Phosphate standard curve was prepared using the Phosphate standard solution supplied in the same kit. Each well in a 96 well plate was loaded with 20 μL of working MG solution. Smooth muscle actomyosin (1 mg/ml) containing 10 mM MgCl_2_ was mixed either with 100 μM CaCl_2_ (activation) or 1 mM EGTA (inactivation) in reaction tubes maintained at 25 °C. Liberation of phosphate was measured after the addition of 1 mM ATP to the reaction mix. Time-point aliquots were taken from each of the ATPase tube at 4 min intervals for 32 min and diluted 50X with ddH2O. 80 μL of this diluted solution was added to the 20 μL MG working solution in the MG solution containing 96 well plates. The plates were incubated for 30 min at room temperature and the absorbance was read at 620 nm in a Spectromax Plus microplate spectrophotometer from Molecular Devices (Sunnyvale, CA). The impact of compounds was determined by incubation of activated or inactivated smooth actomyosin with the compound for 30 min before addition of ATP and P_i_ was quantified as described above.

### 2.3. Time-Resolved fluorescence

Time-resolved fluorescence (TRF) of individual wells in 1536-well plates was measured using a two-channel time-resolved fluorescence plate reader (FLTPR) described in our previous publications [30]. Two TRF waveforms (channel 1 and channel 2) were acquired with each measurement, using bandpass filters (Semrock) that selected two different wavelengths within the donor emission spectrum. The channel 1 measurement was used to determine the fluorescence lifetime of the donor probe by moment calculation according to Eq. (1). The lifetime of the donor probe is decreased by energy transfer to a coupled acceptor molecule. The photophysics of the Alexa-488 Cy3ATP/ADP FRET pair is described in previous publications [2]. The ratio between total fluorescence of channel 1 and channel 2 donor emission (Ch1/Ch2) was used to identify wells containing fluorescence interfering compounds, as described previously [30].


(1) $$\;moment\;=\frac{\sum_{i=n}^{i=m}I_{i}t_{i}}{\sum_{i=n}^{i=m}I_{i}}$$

### 2.4. High-throughput screening

#### 2.4.1. HTS assay

Alexa-488 labeled myosin in high-salt storage buffer was thawed on ice and then centrifuged at 16,000 rcf for 5 min to remove protein aggregates. The supernatant was diluted in high-salt buffer to a concentration of 5 micromolar and then diluted with addition of 100 vol of filament forming assay buffer, mixed by gentle inversion and incubated at room temperature for 30 min. Cy3ATP was added to a final concentration of 1 micromolar, mixed by gentle inversion and the resulting mixture, incubated overnight on ice. During a typical 10-hour daily screening run this reagent was chilled in a 4 °C incubator and protected from light using aluminum foil. The high-throughput screen was run using a specially equipped HighRes Biosolutions automation platform. Along with integrating the FLTPR time-resolved fluorescence reader described above, this robotic system included Echo^®^ 555 (Beckman-Coulter, Inc.) acoustic liquid dispensers, Multidrop Combi (Thermo, Inc.) liquid handlers, and HighRes Biosolutions MicroSpin plate centrifuges and plate incubators. Compounds from the proprietary BMS high-throughput screening library were dissolved in DMSO to a 2 mM concentration and placed in Corning 1536-well Echo^®^ Certified plates (part number 3730). For a typical screening run, 25 nl of compounds to be tested were acoustically transferred into columns 1 through 44 of a Corning 1536-well solidbottom, black, polystyrene plate (part number 3724). On each plate 25 nl of DMSO was transferred into columns 45 and 46 and 25 nl of a 10 mM solution of mavacamten in DMSO was transferred into columns 47 and 48, to enable total signal and background signal, respectively to be determined. Following transfer of DMSO solutions, 5 μl of Alexa-488 labeled myosin with Cy3ATP was transferred into each well using the Multidrop Combi. Assays plates were then incubated at room temperature in a plate incubator for 30 min. Each plate was then centrifuged at 300 x g for 30 s and placed in FLTPR for analysis. Time-resolved fluorescence waveforms were used to compute sample lifetime by moment calculation (Eq. (1)) prior to subsequent analysis.

#### 2.4.2. HTS hit selection criteria

Compounds were selected as Hits for retesting from the pilot screen and primary screens using *a* > 112.5% or ⟨77.5% threshold relative to the median lifetime of wells containing 50 μM Mavacamten. Hit compounds that exhibited interfering fluorescence, detected by ⟩10% deviation of the channel-1/channel-2 donor emission wavelength ratio from the screen median, were not retested.

#### 2.4.3. Assay quality

The robust Z prime (rZ′) assay quality parameters were determined by comparing the lifetime of DMSO control wells on each plate with 50 micromolar Mava positive control wells on each plate computed according to Eq. (2) [31].


(2) $$rZ^{\prime }=1-\frac{3*\left(MAD_{Mava}+MAD_{DMSO}\right)}{|\;MedianFRET_{Mava}-\;Median\;FRET_{DMSO}|}$$

## 3. Results and discussion

### 3.1. Assay development

**Fig. 1.**
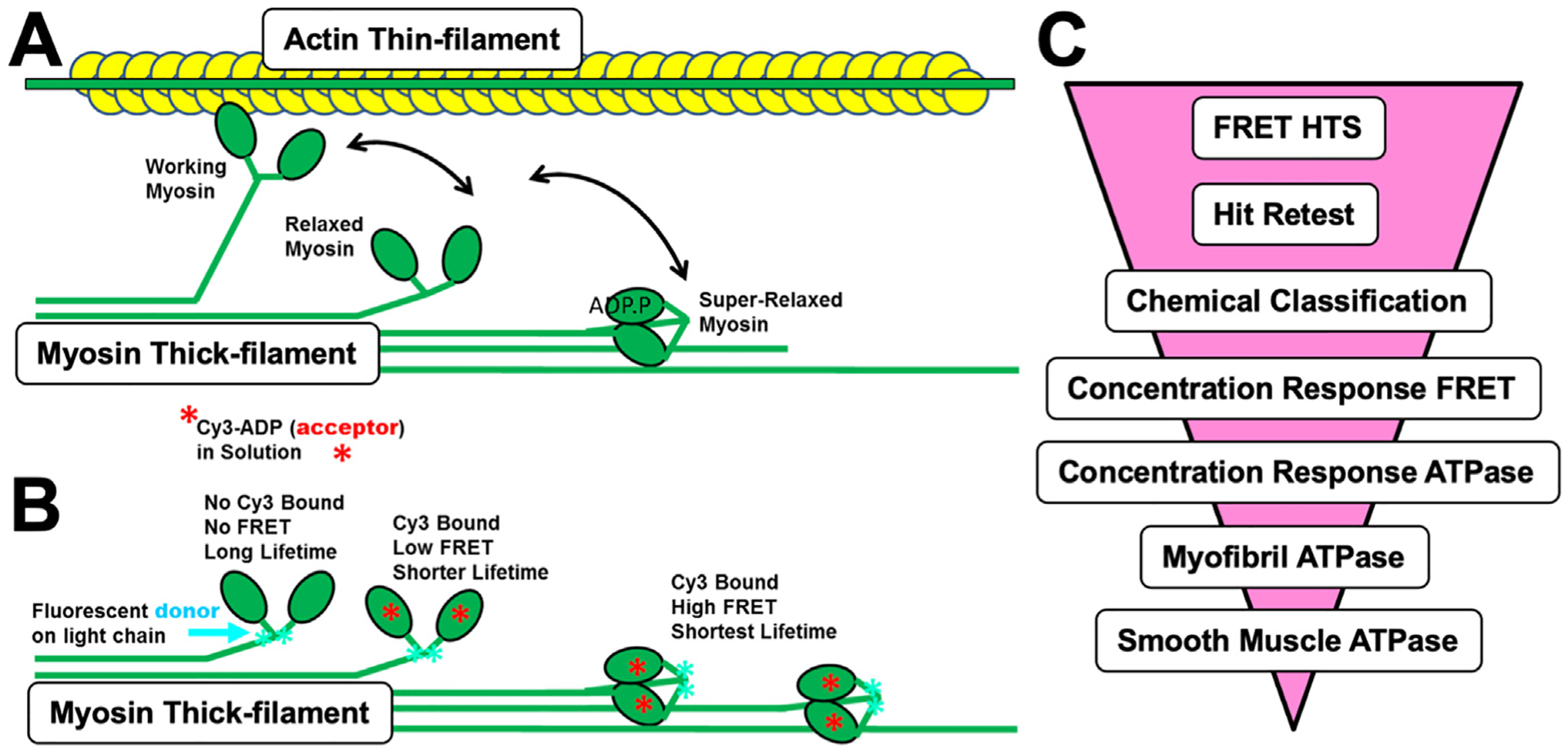
HTS Assay design. (A) Schematic depicting key structural and functional states (Working, Relaxed, Super-Relaxed) of cardiac muscle myosin based on current models of cardiac myosin thick-filament structure and function [18, 40]. (B) Adaptation of a FRET sensor described in detail in Rhode [9] et al. to cardiac myosin synthetic filaments used in HTS. (C) HTS workflow (ordered top to bottom) using the FRET assay to screen chemical libraries (HTS, high-throughput screening).

Our prior work showed that a FRET-based sensor with Alexa-488 labeled cardiac myosin regulatory light chain as a FRET donor and Cy3-ATP or Cy3-ADP as an acceptor detects structural changes induced by OM and Mava binding [9, 12]. Those studies used soluble fragments of myosin that have the filament stabilizing coiled-coil tails removed by proteolytic digestion. In muscle, contractility is facilitated by the myosin thick filament that contains ordered arrays of myosin molecules with their globular N-terminal domains pointed outward toward the actin thin filament (Fig. 1A). The globular N-terminal portion of myosin proteins contains the ATP binding site, the actin-binding interface, and the motor’s light-chain binding domain. In the striated muscle, the motor domain is hypothesized to exist in dynamic exchange between working myosins attached to actin, relaxed myosins extended away from the thick-filament but not attached to actin, and super-relaxed myosins that are bound to the thick-filament back-bone (Fig. 1A) [18]. The structure of the myosin light-chain binding domain changes as myosin transitions between these states as do the energetics of nucleotide binding.

We adapted our soluble myosin FRET sensor assay (Fig. 1B) for a HTS workflow (Fig. 1C) performed on myosin thick-filaments, the fundamental contractile machinery of muscle. The assay is designed to detect Cy3-nucleotide binding via FRET with an Alexa-488 donor attached to the myosin Regulatory Light Chain (RLC) as well as myosin-light chain domain priming in the pre-powerstroke state. We prepared cardiac myosin from bovine heart left ventricles as described in Section 2 Methods. Muscle myosin IIs consist of three proteins, the myosin heavy-chain and two calmodulin-like light chains, the ELC and the RLC. The light-chains can be removed and exogenous light-chains added by mass action under divalent ion chelating conditions. We use this property to attach the recombinant Alexa-488-V105C-RLC described in our previous work [9, 20] to the cardiac myosin light-chain binding domain and then assembled the resulting holoenzyme complex into synthetic myosin thick-filaments [32].

**Fig. 2.**
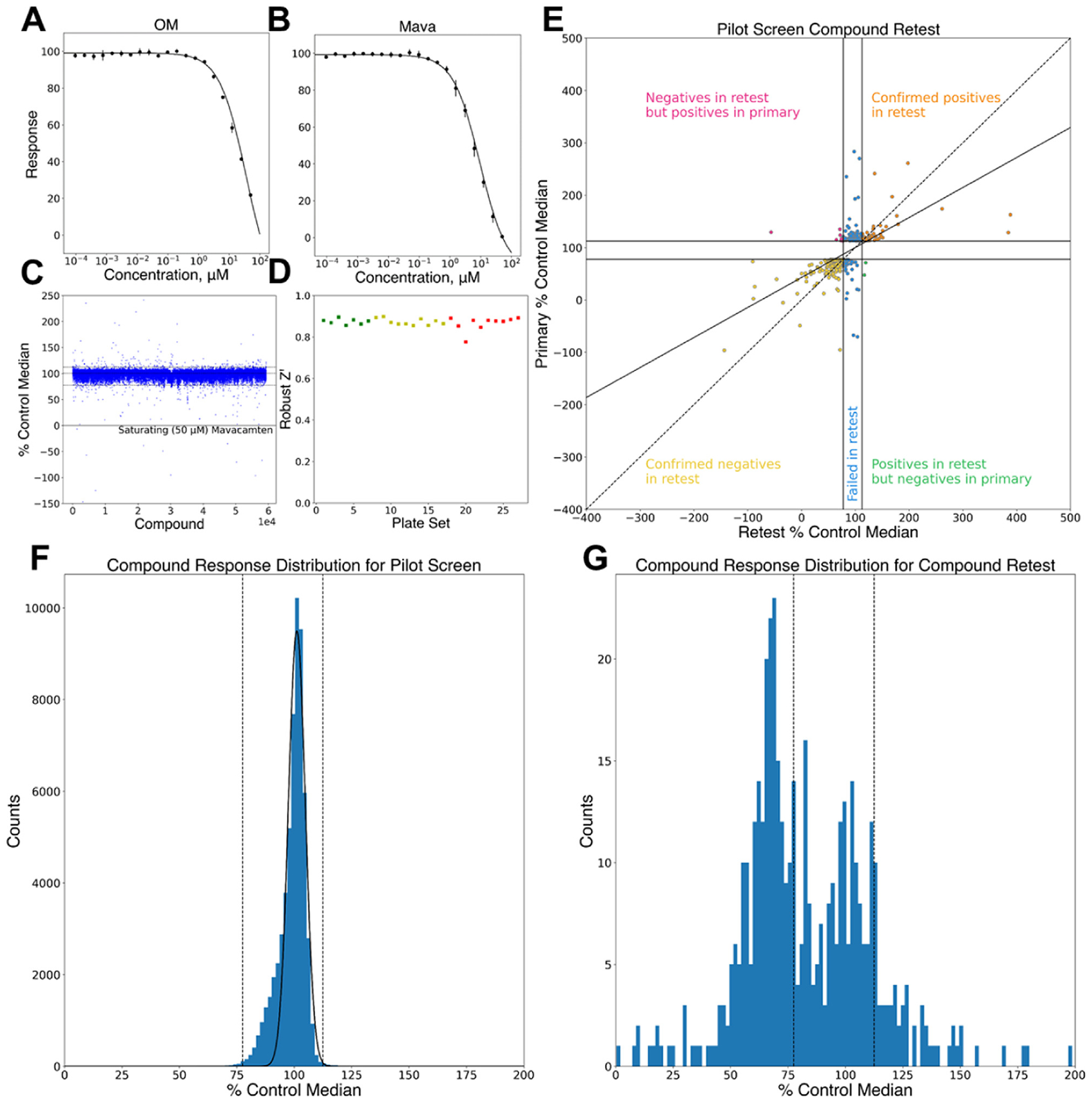
HTS pilot screen. (A) Assay response to control compounds OM and (B) Mavacamten. (C) 60 K pilot screen lifetime. (D) Plate set (colors indicate successive assay runs) robust Z-score mean = 0.87, std = 0.02, min = 0.78, max = 0.90). (E) Pilot screen Hit retest. Confirmed effector compounds localize to upper right and lower left quadrants. Diagonal solid line in panel E indicates linear fit to primary screen Hit delta moment data (Y axis, picoseconds) and Hit retest delta moment data (X axis, picoseconds). Dashed line in panel E indicates slope = 1.0 for reference. (F) Histogram of pilot screen lifetimes. Dashed lines indicate Hit selection thresholds. Solid line indicate Gaussian fit to the histogram. (G) Histogram of the pilot screen retest.

We validated the labeled thick-filaments by measuring the concentration dependence of Alexa-488 to Cy3-ATP/ADP FRET enhancement by OM and Mava (Fig. 2A and 2B). Both compounds induced enhancement of the myosin filament FRET sensor, consistent with their impact on a soluble two-headed myosin preparation [9, 12]. We tested the performance of the assay and readiness for HTS screening by determining the assay’s robust Z-prime scores. Assay material was dispensed in 5 μl aliquots across 1536-well plates in addition to 50 nl of DMSO, or 50 nl DMSO with either OM or Mava. The final reaction concentration of the test compounds was 50 μM. Plates were incubated for 30 min and then the time-resolved fluorescence was read using a two-channel direct waveform recording fluorescence lifetime plate reader (FLTPR) described in Methods. The standard deviation and plate to plate variability of the Alexa-488 donor emission fluorescence were exceptional with the robust Z-prime using 50 μM Mava as a positive test sample > 0.8. These findings provide further evidence that compound mediated FRET enhancement of the myofilament is a useful tool for characterizing both positive and negative allosteric modulators of cardiac actomyosin mechanochemical activities. The adaption of the FRET sensor from the soluble heavy meromyosin format to this form using full-length myosin filament polymers potentially enables detection of additional binding pockets for small molecule allosteric modulators. Furthermore, the high rZ’ observed in high-density microtiter plates suggests that monitoring FLT using this physiologically relevant myosin complex can potentially be useful for screening broad chemical libraries to identify novel myosin modulators.

### 3.2. HTS pilot screen

**Fig. 3.**
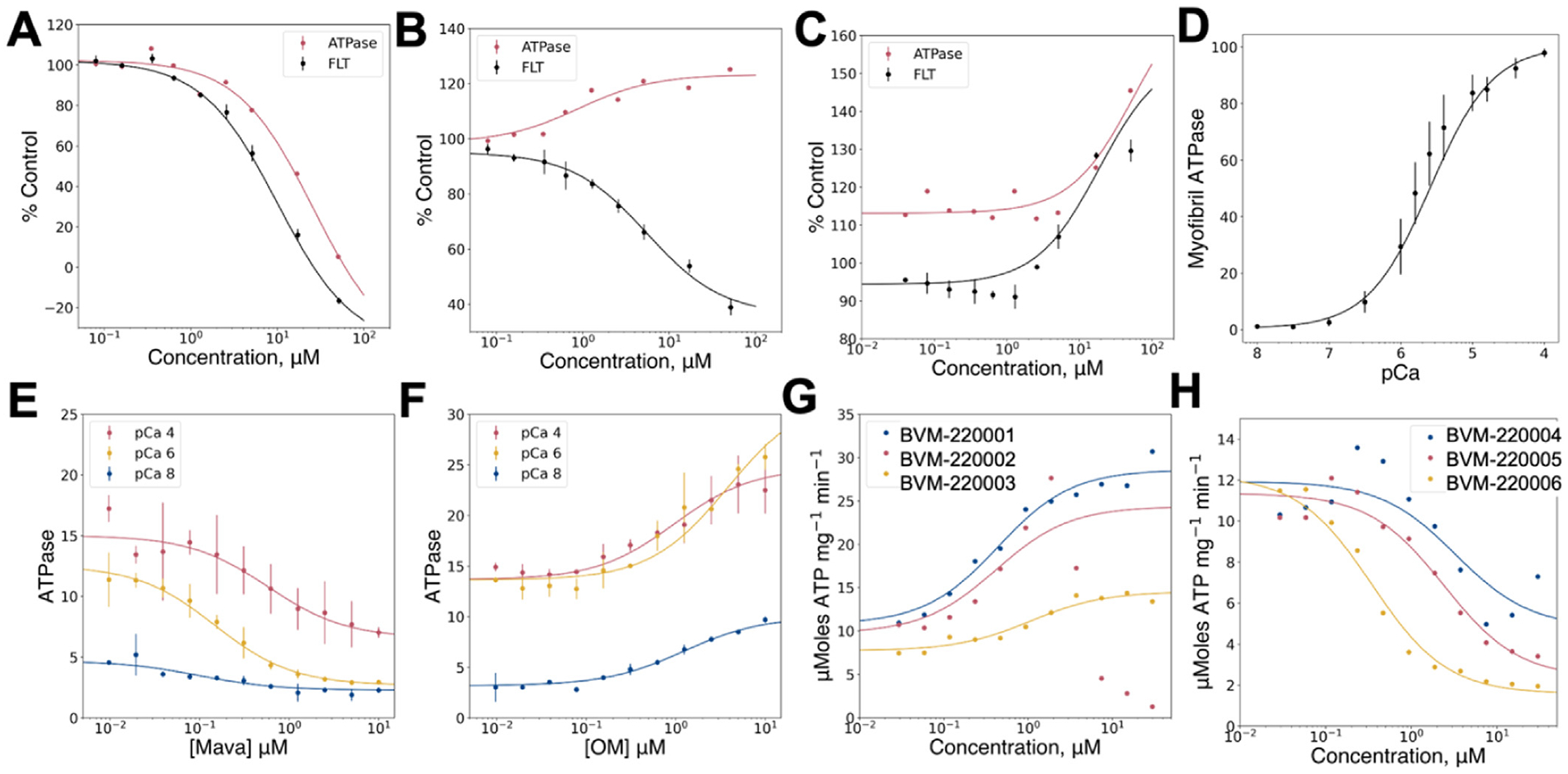
Impact of pilot screen confirmed retest compounds on basal myosin filament ATPase and calcium regulated myofibril ATPase activity. (A, B, C) Compounds separated into three classes based on FRET (black lines and symbols) and myosin filament ATPase activity (red symbols and lines): (A) Increase FRET like Mava and inhibit ATPase activity (B) Increase FRET like Mava and increase ATPase activity (C) Decrease FRET and increase ATPase activity. Myofibril ATPase activity measured at varied concentrations of calcium (D). X-axis pCa is the –log of the Molar free [Ca ^+^ 2]. Myofibril ATPase inhibited by Mava (E) and activated by OM (F). Representative pilot screen myofibril activators (G) or inhibitors (H) tested at free [Ca ^+^ 2] corresponding to 10 ^−4^ Molar (pCa 4.0), 10 ^−6^ Molar (pCa 6.0), and 10 ^−8^ Molar (pCa 8.0).

We optimized the assay and the HTS workflow by performing a 59,362 compound pilot screen (Fig. 2C). Assay mix containing the bovine cardiac myosin FRET sensor at 100 nM with 1 μM Cy3-ATP/ADP, prepared in assay buffer as described in Methods, was dispensed across 1536-well plates containing DMSO alone control wells, DMSO with 50 μM Mavacamten positive control wells, or individual test compounds. The assay reaction plates were incubated for 30 min and then the timeresolved fluorescence of the Alexa-488 donor probe was measured using the FLTPR (Fig. 2C). The screening assay robustly detected compoundinduced changes in time-resolved fluorescence lifetime across the pilot screen. The assay robust Z-prime was consistently > 0.8 for all but one plate set (Fig. 2D). We selected Hit compounds that induced changes in time-resolved fluorescence lifetime using a threshold of > 112.5% the 50 μM Mava control median lifetime (decreased FRET compared to DMSO wells) or < 77.5% the 50 μM Mava control median lifetime (increased FRET compared to DMSO wells). Inside the Hit threshold, the measured lifetimes exhibited a Gaussian distribution which was well approximated by fitting a Gaussian function to the pilot screen data set (Fig. 2F). Outside the Hit selection threshold the lifetimes deviated from the fit Gaussian distribution, consistent with the Hit compounds interacting with the FLT FRET-reporter and perturbing the biochemical conditions of the system. Strongly fluorescent interfering compounds, 24 in total, were identified using the channel-1/channel-2 donor fluorescence emission ratio as described in Hit selection criteria. These interfering compounds were removed from subsequent analysis. Selected Hits totaling 464 compounds were then retested in triplicate. Of these, 279 were confirmed with 57 exhibiting lifetimes > 112.5% the 50 μM Mava (decreasing FRET) and 222 exhibiting lifetimes < 77.5% the 50 μM Mava control (increasing FRET). The observed fluorescence lifetimes of the pilot screen and the pilot screen retest were strongly correlated (Fig. 2E), indicating strong HTS reproducibility. The distribution of lifetimes across the retested samples exhibited a bimodal distribution with false positives that failed retest forming a central Gaussian distribution inside the Hit selection thresholds. Compounds outside the selection threshold significantly outnumbered the false positives consistent with a high degree of retest confirmation. The pilot screen and pilot retest compounds are summarized in Table 1. We measured 10-point concentration response curves (CRCs) for the retest confirmed compounds to determine their 50% effective concentration (EC50). Compounds that passed retest and exhibited CRC EC50 values of less than 20 micromolar were tested for functional impact on basal myosin filament ATPase activity and for functional impact on the calcium regulated ATPase activity of bovine ventricular myofibril ATPase activity (Fig. 3).

Based on the FLT and myosin filament ATPase measurements, the pilot screen Hit compounds were classified into three representative Hit compound groups. The first group decreased myosin filament ATPase activity and decreased lifetime (increased FRET) similar to Mava (Fig. 3A). These compounds are allosteric inhibitors that stabilize a high FRET state while inhibiting ATP turnover. The second class increased ATPase activity and increased lifetime (decreased FRET) (Fig. 3B). These compounds are allosteric activators that stabilize a high FRET state while activating ATP turnover. The third class increased ATPase turnover and increased lifetime (decreased FRET) (Fig. 3C). These compounds are allosteric activators that stabilize a lower FRET state. Compounds that decreased ATPase activity and increased lifetime were deprioritized as they include ATP competitive inhibitors. Hit compounds were tested for impact on myofibril preparations, validated for calcium dependent ATPase activation over a range of activating conditions (Fig. 3D) and in response to existing control compounds Mava (Fig. 3E, allosteric inhibitor) and OM (Fig. 3F, allosteric activator). Several of the Hit compounds were robust myofibril activators (Fig. 3G) and inhibitors (Fig. 3H). Taken together, the pilot screen demonstrated the utility of our FLTPR FRET screening approach for identifying compounds that act as allosteric activators and inhibitors of cardiac myosin in muscle preparations. Successful completion of the pilot screen prompted a larger efArticle 03: Untitled fort deploying the assay workflow in a 1.6 million compound HTS campaign.

### 3.3. Primary hts

**Fig. 4.**
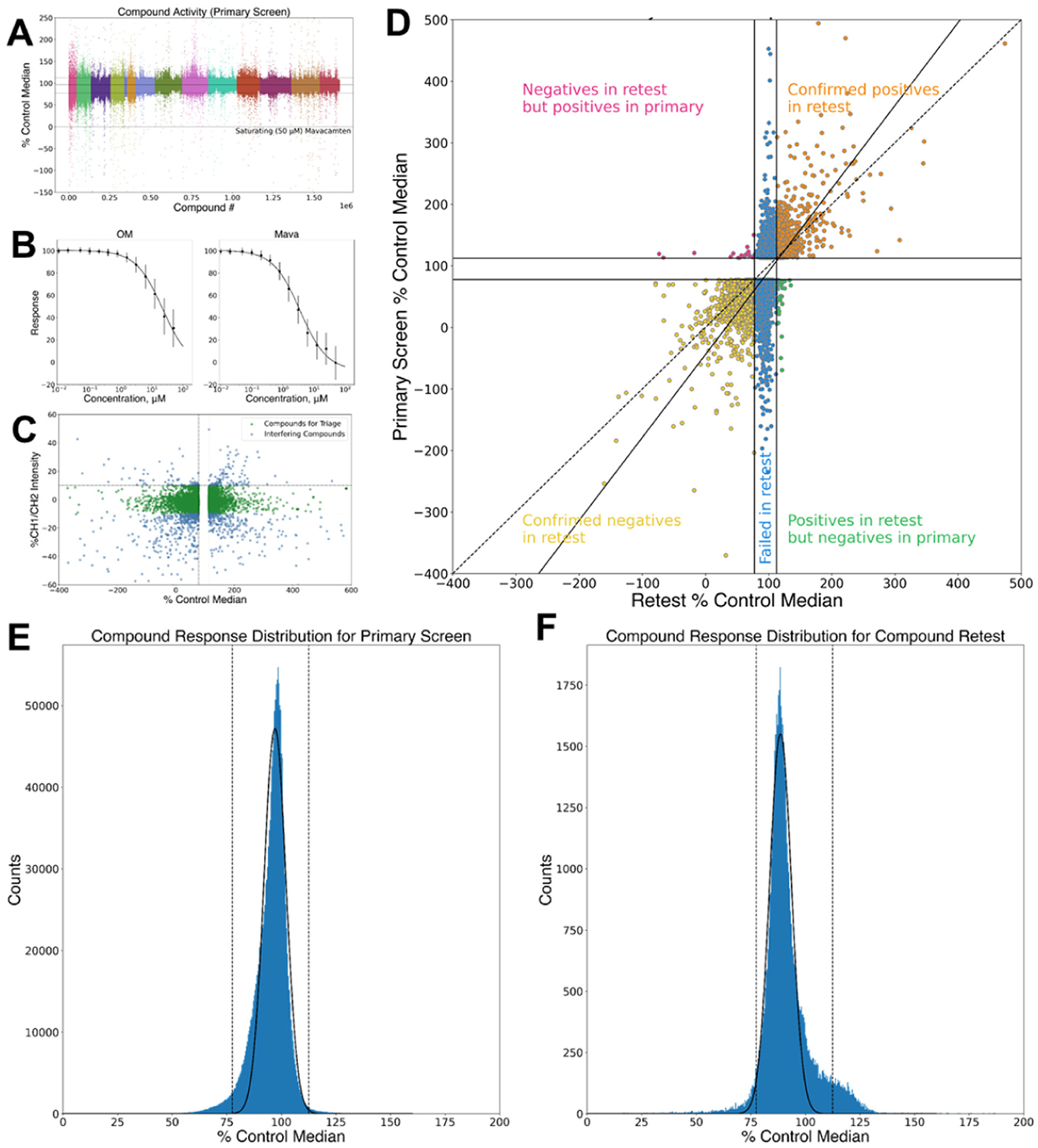
Automated HTS. (A) Assay moment robust Z-score for 1.6 M compound library screen. Colors indicate independent assay runs performed on different days. (B) Mean response of the FLT assay to OM and Mava +/− standard deviation of control compounds during the 1.6 M compound library screen. (C) Fluorescence interference filter CH1/CH2 intensity ratio (y-axis) vs. FLT% Mava Control Median. Compounds that exceeded 10% increase and decrease (blue) in the CH1/CH2 ration (horizontal lines) were deprioritized. Compounds within this range that exhibited FLT > 112.5% or < 77.5% Mava control median where advanced for retest. (D) Hit retest primary screen FLT relative control median compared to retest FLT relative Control Median. Confirmed effector compounds localize to upper right and lower left quadrants. Diagonal solid line in panel D indicates linear fit to primary screen Hit change in moment relative to the median control (Mava) change in moment (Y axis) and Hit retest change in moment relative to the median control (Mava) change in moment (X axis). Dashed line in panel D indicates slope = 1.0 for reference. (E) Histogram of primary screen lifetimes. Dashed lines indicate Hit selection thresholds. Solid line indicates Gaussian fit to the histogram. (F) Histogram of the pilot screen retest with thresholds and Gassian fit line as described for panel E.

For the 1.6 M compound screen, we prepared assay mix fresh each day prior to initiating screening following the same protocol optimized for the pilot screen. DMSO only conditions and samples with 50 μM Mava were included in each plate for the plate-to-plate determination of assay response and Z-prime. The performance of the assay in the 1.6 million compound screen was comparable to the pilot screen (Fig. 4). The average robust Z-prime exceeded 0.8. We measured the donor fluorescence lifetime at two emission wavelengths and used the ratio of the total fluorescence at these wavelengths to infer fluorescent compound interference and to deprioritize these interfering compounds as likely false positives [30]. Hit compounds were selected using a threshold of > 112.5% 50 μM Mava control median lifetime or < 77.5% 50 μM Mava control median lifetime. We evaluated the distribution of compound fluorescence lifetimes across the primary screen (Fig. 4E). The majority of compounds that did not exceed the Hit selection criteria exhibited characteristics of a Gaussian distribution. Beyond the Hit thresholds, the distribution deviated from a Gaussian distribution. This is expected and is the basis of the Z ′ HTS assay performance metric [31]. In the FLT assay, the distribution of lifetimes of non-effector wells should be randomly distributed. Effector compounds that change the assay FLT do so by changing the biochemical conditions of the assay system which thus deviate from the random distribution of non-effector wells.

### 3.4. HTS hit retest and functional validation

**Fig. 5.**
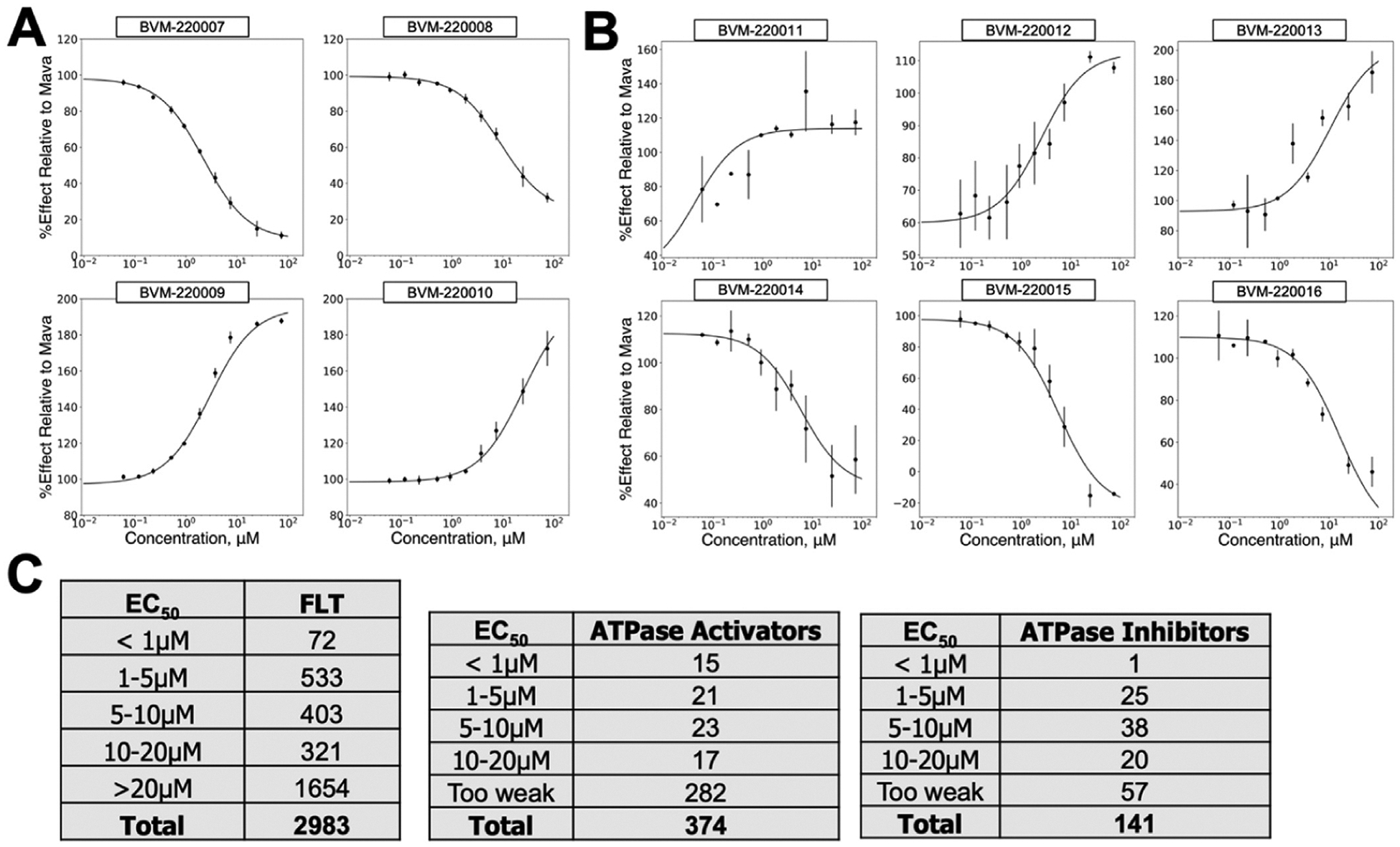
Myosin Filament FLT and ATPase CRC of retest positive compounds. (A) Representative FLT decreasing (top, similar to Mava) and increasing (bottom) compounds. (B) Representative filament ATPase activators (top) and inhibitors (bottom). (C) Summary of EC50 values from CRC analysis.

A total of 62,512 compounds were selected for retest in triplicate (Table 1). The selection was based on their impact on the fluorescence lifetime of the FRET sensor and their minimal impact on the channel1/channel-2 fluorescence intensity ratio (Fig. 4C). Of the Hits, 13,402 (20%) decreased FRET while 49,970 (80%) increased FRET, similar to Mava. Following retest, 6230 compounds reproduced with 3731 (60%) decreasing FRET, and 2499 (40%) increasing FRET. As in the pilot screen, the distribution of fluorescence lifetimes from retest compounds exhibited a characteristic Gaussian distribution inside the Hit threshold window and deviated from this distribution outside the Hit selection threshold (Fig. 4F). Differences in the relative number of false positives in the pilot screen and primary screen reflect differences in the composition of the two libraries. We segregated Hit compounds into distinct chemical classes and selected 3000 compounds with representatives from each chemical class for concentration response curve (CRC) testing using the primary FRET assay to determine each compounds 50% effective concentration and apparent maximum efficacy. Representative compounds from these tests are shown in (Fig. 5). Of the 3000 CRC tested compounds, 605 exhibited EC50 CRC response values lower than 5 μM, 72 of these were less than 1 μM.

**Fig. 6.**
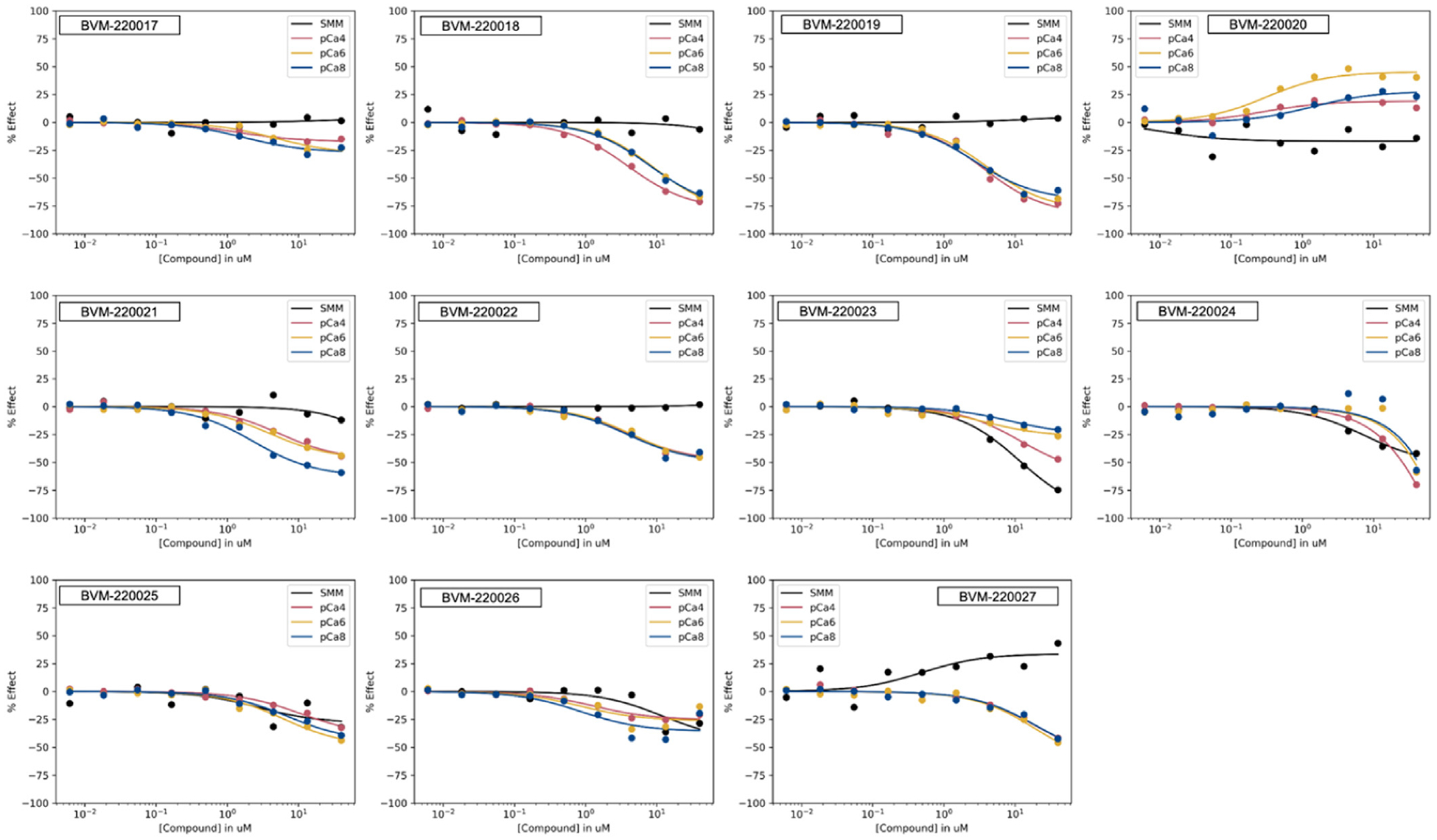
Cardiac Myofibril ATPase measured at pCa 4.0, 6.0, and 8.0 and Aorta Smooth Muscle ATPase (SMM) activity of select allosteric modulators.

Compounds with FRET CRC EC50 values below 20 micromolar were evaluated for effects on basal myosin filament ATPase activity (Fig. 5B). A small set of select compounds with impacts on the FRET assay and on myosin filament ATPase activity were tested for impact on cardiac myofibril ATPase activity (Fig. 6) measured at low (pCa 8.0), intermediate (pCa 7.0), and high (pCa 4.0) free Calcium. Compounds were also tested on a preparation of Aorta smooth muscle, enriched in smooth-muscle myosin to verify cardiac myosin II engagement (Fig. 6) but not engagement of smooth-muscle myosin.

## 4. Conclusions

Our results validate industrial-scale use of a direct waveform recording FLT-FRET approach to small-molecule discovery and demonstrate the potential of this methodology to identify allosteric inhibitors and activators of cardiac myosin. A single FLT-FRET HTS campaign identified both activators and inhibitors that significantly affected ATPase activity of purified cardiac myosin and isolated cardiac myofibrils. These results justify (1) future medicinal chemistry efforts to improve potency and specificity of lead compounds and (2) continued application of the approach to other protein targets including those that are relevant to treatment of heart disease [15, 33]. Myosins have emerged as an intriguing drug target for a variety of diseases. Compounds that target cardiac myosin, including control compounds OM and Mava used in this study, are the best examples. Both compounds alter allosteric structural changes associated with force generation and activation. Other myosin family members are also promising drug targets. These include, fast skeletal muscle myosin [34], non-muscle myosin IIs [35, 36], and plasmodium myosins [18]. The latter provide a unique and selective way to kill parasites such as malaria. For many of these targets, inhibitors are desired. However, with cardiac myosin, both activators and inhibitors are desired. Inhibitors such as Mava, which was recently approved by the FDA [37, 38], are sought to reduce contractility in hypertrophic cardiomyopathies and diastolic dysfunction. Activators such as OM are sought to increase contraction in systolic heart failure [39]. The activators and inhibitors discovered in this screen are examples of leads in both classes, thus demonstrating the ability of a single, structure-directed FLT-FRET screen to identify compounds with structural impacts on the target, and also distinct impacts on protein function, in this case cardiac myosin ATPase activity and myofibril ATPase activity. A valuable outcome of our work is the demonstration of a nanosecond time-resolved fluorescence lifetime FRET measurement being deployed in a successful HTS campaign in an demanding, highly standardized, industrial setting.

## Figures and Tables

**Table 1:** Compound selection summary. Number of compounds that pass each stage of the screening workflow in the pilot and primary screens.

	Pilot Screen	Primary Screen
Initial Compound Pool	59,362	1,670,481
> 112.5% Control Median	221	13,402
< 77.5% Control Median	300	49,970
Interfering Compounds	24	860
Total Hits	497	62,512
Hit Frequency	0.84%	3.74%
Retested Compounds	464	59,905
Retest >112.5% Control Median	57	3731
Retest <77.5% Control Median	222	2499
Confirmed Hits	279	6230
